# Using visual art and collaborative reflection to explore medical attitudes toward vulnerable persons

**Published:** 2016-03-31

**Authors:** Monica Kidd, Lara Nixon, Tom Rosenal, Roberta Jackson, Laurie Pereles, Ian Mitchell, Glenda Bendiak, Lisa Hughes

**Affiliations:** 1 Department of Family Medicine, University of Calgary; 2 Department of Critical Care Medicine, University of Calgary; 3 Department of Pediatrics, University of Calgary; 4 Department of Classics and Religion Faculty of Arts, University of Calgary

**Keywords:** Reflective practice, vulnerable populations, visual art, body image

## Abstract

**Background:**

Vulnerable persons often face stigma-related barriers while seeking health care. Innovative education and professional development methods are needed to help change this.

**Method:**

We describe an interdisciplinary group workshop designed around a discomfiting oil portrait, intended to trigger provocative conversations among health care students and practitioners, and we present our mixed methods analysis of participant reflections.

**Results:**

After the workshop, participants were significantly more likely to endorse the statements that the observation and interpretive skills involved in viewing visual art are relevant to patient care and that visual art should be used in medical education to improve students’ observational skills, narrative skills, and empathy with their patients. Subsequent to the workshop, significantly more participants agreed that art interpretation should be required curriculum for health care students. Qualitative comments from two groups from two different education and professional contexts were examined for themes; conversations focused on issues of power, body image/self-esteem, and lessons for clinical practice.

**Conclusions:**

We argue that difficult conversations about affective responses to vulnerable persons are possible in a collaborative context using well-chosen works of visual art that can stand in for a patient.

## Introduction

Open discussion around health care providers’ affective responses to vulnerable persons can be difficult using traditional pedagogy rooted in positivist, biomedical explanation of disease.^1^–^3^ Yet these discussions are critical to help reduce stigma-related barriers vulnerable persons face while seeking care.^4^–^10^ Visual arts are used increasingly in health care education to improve students’ observational and diagnostic skills.^11^–^16^ Emerging research suggests health care students not only gain important clinical skills from such teaching, but also that arts experiences help develop expressive capacity, enhance attitudes (e.g. engagement with the concept of empathy), and support professional development.^17^–^19^ More importantly, the visual arts may challenge clinicians’ assumptions about patients and patterns of automatic thinking by making them uncomfortable.^20^ More than half of North American medical schools now use visual arts to achieve curriculum goals.^21^

We wanted to build on these trends and create a visual arts professional development tool at the University of Calgary that would allow us to explore issues around vulnerable persons, because of the difficulties doing this with traditional teaching approaches. We chose a discomfiting painting that we believed would generate conversation around power, gender, body image, and medical labeling. We then designed a workshop to promote deep thinking and reflection^22^,^23^ in two ways: to use group observation and Visual Thinking Strategies^24^ to lead medical viewers into an image more deeply than they may have gone on their own; and to collaboratively interrogate the group’s ideas about vulnerability in the micro- (personal), meso- (community), and macro- (professional) spheres.^25^ We chose a collaborative approach for two reasons. First, we wanted to build on our institution’s history with group problem-based learning pedagogy. Second, because the arts often challenge positivistic ways of knowing, and those in health care can be resistant toward notions that “truth” is socially constructed,^26^ we felt a collaborative approach might make the experience more accessible. We have run the workshop three times in three different professional development contexts, with slight variation each time based on experience from the previous workshop. We present individual and group responses from two iterations for discussion here.

The centerpiece of the workshop is a case study of Margaret Sutherland’s oil on linen painting, *Sticky*. We focus on the theme of the unclothed female body, as well as presenting some of the formal elements used in art. Ours is a departure from traditional approaches to teaching art in medical education, which typically involve exposing participants to a variety of art genres and media to fine-tune observation and pattern recognition skills.^27^,^28^ Although assumptions may be explored and multiple interpretations acknowledged in more traditional approaches, interpretation is often framed in objective terms (what can be seen by all); the body is viewed largely as object with disease, in keeping with Martin Buber’s “I-It relationships,” based on abstraction, causality, detachment, and utility.^29^ An inherent gap in these methods is the reluctance to engage with the body (and patients) per se – the body as subject, with specific unique experiences, not being the focus of traditional approaches. I-Thou relationships, “characterized by spontaneity, subjectivity, reciprocity, and recognition and acceptance of the unique other” are not fostered.^29^ Our collaborative approach, in contrast, invites participants to re-engage with the body and the individual’s unique experience which said body represents.

## Methods

**Figure 1:**
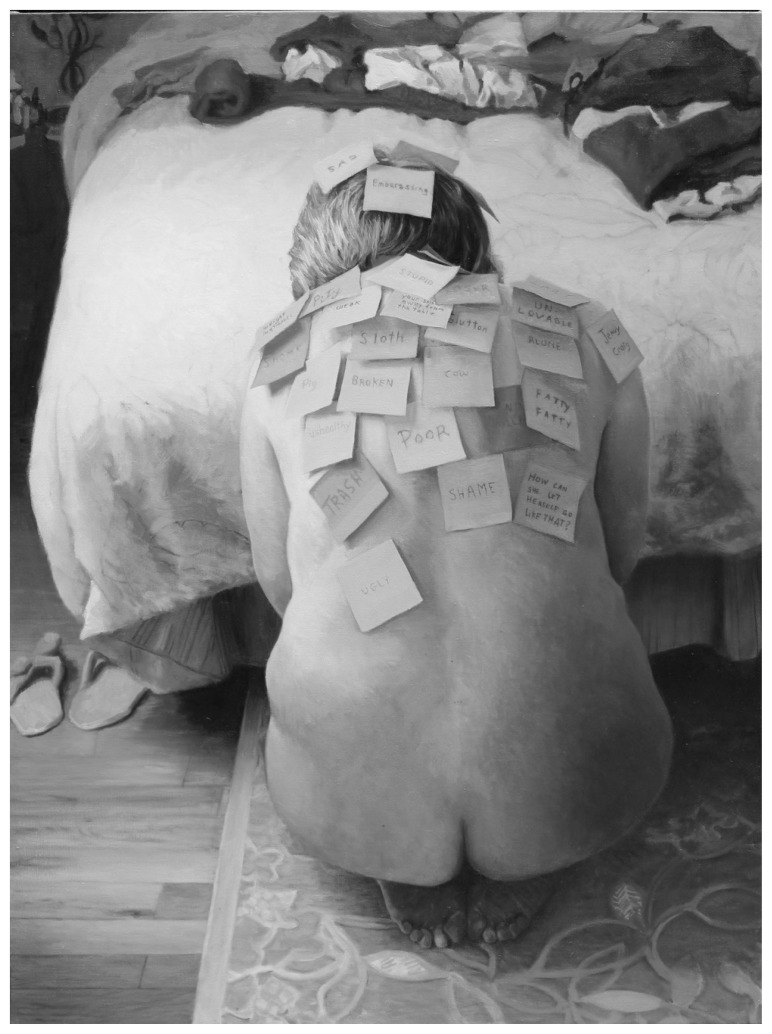
*Sticky*, by Margaret Sutherland (2012) 40 inches by 60 inches, oil on linen, private collection. Used with permission from the artist. Color version available from: http://maggiethered.com/images/Sticky.htm.

The centerpiece of the “Sticky Questions” workshop is the 2012 painting *Sticky* (40-inch by 60-inch oil on linen) by Kingston, Ontario-based portraitist Margaret Sutherland (Figure 1). The painting features an unclothed grey-haired woman, seen only from behind, kneeling at the foot of a bed. Her head and torso are covered with sticky notes declaring insults and labels such as *Sloth, Broken, Poor, Trash*. Our first workshop, planned as a one-hour session for a group of health care professionals (HCP), health research scholars, and health care students, was held during a symposium on humanities in health care in Calgary in March 2013.

Participants heard introductory remarks from a Classicist trained in Art History (LH) on how to “read” a work of visual art. The instructor presented the traditional formal elements (medium, line, color, texture, and space) that art historians use to analyze and interpret art. In order to elaborate on these elements the participants viewed representative images of unclothed female bodies, with particular emphasis on examples where the gaze of the subject is obscured from the viewer as this was a key element in our focal art piece.

*Sticky* was then unveiled, and participants were given a few minutes to view the painting on their own. They were encouraged to note down images and questions the painting raised for them on a pad of sticky notes provided for this purpose. We asked them, when possible, to frame their responses as questions rather than as declarative statements in order to emphasize the ambiguity involved in the act of viewing. We tried to create a micro-context that encouraged participants to “use everyday diplomacy [and] dialogic conversation” to invite an array of (potentially conflicting) opinions.^30^

Following approximately five minutes of independent viewing, participants were divided into four facilitated groups in which they were asked to share their ideas and questions. Facilitators used the techniques of Visual Thinking Strategies^24^ to lead participants deeper into the image by asking three basic probing questions: What’s going on in the image? What makes you say that? What else can you see? In this way, participants were encouraged to articulate their impressions by providing evidence from the painting itself. Facilitators attempted to frame and link participant responses in a neutral manner. Similarly, participants were asked to respect each others’ responses. Groups recorded their findings on flipcharts. After about 10 minutes of small group work, participants reconvened in the large group for a further 10 minutes of large group discussion. The workshop concluded with a 10-minute question-and-answer period with the artist, who was in attendance.

At the conclusion of the workshop, we collected the participants’ written work (flipcharts and anonymous sticky notes) for thematic review. Eight weeks after the workshop, we emailed participants questionnaires inviting comment on the mechanics and content of the workshop as well as any impact they felt the workshop had had. The post-workshop questionnaire was performed in order to broadly assess participant satisfaction, and gather suggestions to improve the workshop in the future, once the participants had had the opportunity to reflect on the experience. This assessment included ranking and commentary on the various sections of the workshop, as well as a retrospective pre-post self-assessment of participants’ attitudes towards the value of art in medical education. Changes in self-assessed attitude were analyzed using the Wilcoxon signed-rank test.

Based on feedback from our first workshop, we subsequently lengthened the workshop from one hour to 90 minutes, providing more time for small group work and less time for large group discussion. We conducted this adapted workshop for a national audience of family physicians in Vancouver in November, 2013. For this workshop, the artist made herself available for a question and answer period via Skype. Similar to the first workshop, we collected flipcharts and anonymous sticky notes for analysis, but we did not conduct the post-workshop questionnaire. These activities were granted ethics approval by the Conjoint Health Research Ethics Board (ID 25240).

## Results

### Pre-/post quantitative analysis

Twelve of the 20 participants in the first workshop responded to the post-workshop questionnaire (60% response rate). Of these, 92% (11/12) rated the workshop as valuable, and significant changes in several self-assessed attitudinal measures were observed (Table 1). Participants’ comfort level with interpreting visual art significantly improved following the workshop. Participants were somewhat more likely after the workshop than before to agree that there are multiple ways to interpret visual art and that professional identity can affect this interpretation, though these changes were not significant. There was very high agreement with the statement “cultural background, values, and beliefs can affect how people interpret visual art” prior to the workshop (4.6 out of 5), so no change was observable here.

Participants felt significantly more strongly after the workshop that (i) the observation and interpretive skills involved in viewing visual art are relevant to patient care; and (ii) visual art should be used in medical education to improve students’ observational skills, narrative skills, and empathy with their patients. Finally, participants were more likely after the workshop than before to endorse the statements “art interpretation should be taught electively to health care students,” and significantly more participants agreed that, “art interpretation should be required curriculum for health care students”.

To summarize the quantitative results, after guided discussion of *Sticky* participants felt more strongly that the skills they used in interacting with visual art are relevant to patient care and should be used in medical education.

### Qualitative responses

Though participants in the two workshops comprised very different groups of medical viewers, both responded to *Sticky* in similar ways, so we combined their observations for review.

Many of the participants’ responses were point-form, so an in-depth thematic analysis of individual reactions is not possible, but neither is it desirable, given our collaborative approach to “reading” the painting. We did not record the sessions, as we did not want to interfere with the free flow of conversation; however as we attended the workshops, we are able to comment on general themes raised during our discussions about the painting. Responses fell into the following categories: power, body image/self-esteem, and lessons for clinical practice.

### Power

Viewers commented on how the subject’s position — somewhere between kneeling and rising, with her face turned away — suggested “overwhelming vulnerability” and a “crushing sense of hardship, shame and humiliation.” They wondered why she was depicted in that position, and whether it represented “prayer, supplication or resignation.” Religious interpretations were also evident in how some viewers interpreted comments on the sticky notes covering the woman back as deadly sins. Common responses were to wonder why the sticky notes were placed where they were, and who had chosen to put them there; one participant specifically wondered whether the notes were applied before or after whomever applied them got to know her. One participant commented on how the scene reminded him of torture images from the United States Abu Ghraib prison in Iraq. These comments reflected in-depth discussion around ideas of power between the subject and a presumed oppressor (external or internal). The viewer, as participant in the subject’s distress, is implicated in whatever violation of power may have taken place; the viewer who is also a HCP is left to question how the subject (patient) may be left marked by the viewing (clinical encounter).

### Body image/self-esteem

Many of the comments about body image and self-esteem picked up on the discordance between the subject’s appearance and the harsh labels applied to her, remarking that the subject appeared only “pleasantly plump,” and not deserving of the labels applied to her. There was also tension between the tone of the comments on her back and the “tidy, beautiful room,” as though a person who truly represented the slurs written on the subject’s back would be expected to live in squalor, whereas the subject appeared “well-to-do to upper middle class because of the flooring, clothes and haircut.” Not all participants were comforted by the subject’s context, however; one commented that she considered the palette “disturbing” and wondered if the subject were, in fact, dead, as what living person would allow someone to do something like that to her?

Other comments focused on the depth or superficiality of the insults: one viewer felt the insults lurked somewhere under her clothes and therefore “clothes don’t hide her from judgment,” whereas another viewer felt because the “sticky notes don’t penetrate the skin” that “they have been applied to her but can be removed.” Another participant went one step further and wondered what might happen if the sticky notes were simply removed. In general, HCP seemed challenged by the content of the sticky notes, and worked to reconcile the dissonance between their “objective” observations and the “subjective” picture presented by the content of the sticky notes.

### Lessons for clinical practice

Participants were quick to take lessons for their own clinical practice from group conversations with other professionals about *Sticky*. For most, the sticky notes were an obvious metaphor for medical labeling; one participant commented, “By labeling people we set them apart from us, dehumanize them.” Not all participants endorsed the idea, however, as many argued that labels are often quite useful shortcuts, and as far as the painting was concerned, the superficial nature of the sticky notes showed that labels are not permanent. Several responses suggested participants endorsed the possibility that “truth” is socially constructed. One participant questioned whether his gender might affect his interpretation of the subject and her circumstance. Another participant said, “We project ourselves and our own worldview onto the painting;” while another with formal art history training commented, “We bring our past knowledge of viewing art and our artistic preferences as well as our lens to interpreting *Sticky*. On some level, we interpret all paintings in the same way,” begging the question whether the same might be said of HCP “interpreting” patients.

## Discussion

These facilitated small-group discussions with health care professionals about the painting *Sticky* provided rich shared experiences, and an opportunity to discuss uncomfortable issues around vulnerability and power in medicine. Using a piece of art to spur conversation rather than the traditional medical case-based discussion allowed groups to approach these difficult issues obliquely, without blame or too much focus on specific scenarios. Reflection on comments made during the workshops leads us to three general conclusions about HCP’s responses to the Sticky Questions workshop: (i) the painting sensitized them to the ways in which patients may be left marked by the viewing/clinical encounter; (ii) the workshop left them discomfited by discrepancies between others’ interpretations and their own “read” of a situation, and they were motivated to understand the positions of others; and (iii) they endorsed the idea that medical “truth” — their read of a painting, or their assessment of a patient — could be authored in part by their own gender or worldview, and that these personal characteristics of the viewer might in fact color all of his/her interactions with the world.

Authentic discussion of our attitudes, values, and experiences in medical education is challenged on many fronts. It is seen by some as “soft” curriculum in contrast to “core topics” which relate to disease processes, their diagnosis and treatment. Even in the teaching of communication and physical examination, skills development is the focus with emphasis on objective observation and data gathering. Our own subjective experience of others is not typically discussed. Exploration of how to approach the body or engage with vulnerability, our own or that of our patients, is anything but routine.

In fact, during training, physicians are taught strategies to desexualize physical examination, including objectifying patients’ bodies.^31^ These efforts to suppress inappropriate sexual feelings towards our patients and maintain professional objectivity risk broad desensitization. Weller alludes to this and challenges “the medical gaze” with the work of Frida Kahlo, who “revolutionized art for women, by depicting her body as it was…express[ing] the experience of [her own body], rather than being depicted as the passive sexualized object of the male gaze” or the desexualized object of the medical gaze.^28^ Margaret Sutherland’s *Sticky* provides a similarly “startlingly honest image” of her experience of her body; *Sticky* invites viewers to engage deeply and thoughtfully, as our participant’s responses support.

These observations lead us to believe that participants “had an experience,” in the Deweyian sense.^32^ Philosopher John Dewey argued that experience — an emotional and sensory adventure one participates in that requires active engagement, and rewards with a sense of completeness, closure, and reconstruction — moves a person from fixed recognition to more mature perception: “In recognition, we fall back, as upon a stereotype, upon some previously formed scheme…[T]o perceive, a beholder must *create* his own experience.” Further: “The one who is too lazy, idle, or indurated in convention to perform this work will not see or hear. His ‘appreciation’ will be a mixture of scraps of learning with conformity to norms of conventional admiration.” Dewey was referring here to participating in art, but he could have easily been speaking of medical diagnosis and treatment.

These findings suggest that difficult conversations about affective responses to vulnerable persons are possible in a collaborative context using well-chosen works of visual art that can stand in for a patient. It is consistent with recent psychological research showing that engagement with fictional characters can bolster our own identity and improve empathy,^33^ and that being curious about others is an exercise in the collective meaning-making necessary for sociality,^34^ both of which, we would argue, are necessary for professional behavior.

This study, like many other investigations of the effectiveness of medical humanities “interventions,”^35^ suffers from two main design flaws: participants who took part in the workshops did so voluntarily so the workshop may have only “preached to the converted,” and we did not have a control group. Similarly, those who chose to complete the retrospective pre-post attitudinal questionnaire may have felt more positively towards the project as a whole, as compared to the group who did not return the survey. This may also have limited our ability to identify pre-post differences, given that we anticipated that these responders would rate their attitudes towards the importance of art and the medical humanities relatively high at baseline. For this reason, we had chosen to perform a retrospective pre-post attitudinal questionnaire (as opposed to a traditional pre-post attitudinal questionnaire), as retrospective assessments have been shown to be more sensitive in situations where training is expected to influence participants’ criteria for self-ratings by creating awareness of their gaps in knowledge, although by nature of the timing of administration, they may be more susceptible to recall bias.^36^,^37^

Furthermore, while our limited questionnaire data suggest the workshop may have changed one group’s attention to their own perception, we cannot conclude that anyone’s *behavior* toward vulnerable persons (the desired outcome) necessarily changed. We also had small group sizes (though larger sizes may have impaired the intimate discussions we were able to have in small groups), and most of the responses available to us for comment were point-form, therefore in-depth thematic analysis was not possible, as it might have been if we had held focus groups or follow-up interviews.

In spite of these limitations, we believe the “Sticky Questions” workshop builds skills for two moments of the clinical encounter:^38^ attention (taking the history and developing the therapeutic relationship); and representation (integrating, charting and referring). Though we have been impressed with the power of *Sticky* to generate important conversations, we also believe the process we have established could be used with other works of visual art. In the future, we hope to build on the workshop by incorporating before- and after-participation written reflection to help us assess whether an art-based discussion of the perceptions of vulnerable persons by HCPs persist beyond the workshop.

## Figures and Tables

**Table 1:** Participant mean self-assessment of attitudes towards the value of art in medical education, retrospective pre- and post-workshop.

	Mean Pre	Mean Post	*p*-value*
I am comfortable interpreting visual art.	3.0	3.8	<0.05
There are multiple ways to interpret visual art.	4.3	4.7	NS
Cultural background, values, and beliefs can affect how people interpret visual art.	4.6	4.7	NS
Professional identity can affect how people interpret visual art.	4.1	4.6	NS
The observation skills involved in viewing visual art are relevant to patient care.	3.5	4.7	<0.05
The interpretive skills involved in viewing visual art are relevant to patient care.	3.3	4.4	<0.05
Visual art should be used in medical education to improve students’ observational skills.	3.5	4.6	<0.01
Visual art should be used in medical education to improve students’ narrative skills.	3.2	4.2	<0.01
Visual art should be used in medical education to improve students’ empathy with their patients.	3.4	4.5	<0.01
Art interpretation should be taught electively to health care students.	3.1	3.7	NS
Art interpretation should be required curriculum for health care students.	2.9	3.5	<0.05

Scale: 1 = strongly disagree, 5 = strongly agree. Greater agreement is equivalent to more positive attitudes towards the value of art in medical education. NS = not significant.

* Wilcoxon signed-rank test, paired, two-tailed.
